# Comprehensive molecular and cellular characterization of endoplasmic reticulum stress-related key genes in renal ischemia/reperfusion injury

**DOI:** 10.3389/fimmu.2024.1340997

**Published:** 2024-03-01

**Authors:** Hao Zhang, Chaoyue Zheng, Yue Xu, Xiaopeng Hu

**Affiliations:** ^1^ Department of Urology, Beijing Chao-Yang Hospital, Capital Medical University, Beijing, China; ^2^ Institute of Urology, Capital Medical University, Beijing, China

**Keywords:** renal ischemia-reperfusion injury, endoplasmic reticulum stress, renal transplantation, single-cell gene expression analysis, bioinformatics

## Abstract

**Background:**

Renal ischemia-reperfusion injury (RIRI) is an inevitable complication in the process of kidney transplantation and lacks specific therapy. The study aims to determine the underlying mechanisms of RIRI to uncover a promising target for efficient renoprotection.

**Method:**

Four bulk RNA-seq datasets including 495 renal samples of pre- and post-reperfusion were collected from the GEO database. The machine learning algorithms were utilized to ascertain pivotal endoplasmic reticulum stress genes. Then, we incorporated correlation analysis and determined the interaction pathways of these key genes. Considering the heterogeneous nature of bulk-RNA analysis, the single-cell RNA-seq analysis was performed to investigate the mechanisms of key genes at the single-cell level. Besides, 4-PBA was applied to inhibit endoplasmic reticulum stress and hence validate the pathological role of these key genes in RIRI. Finally, three clinical datasets with transcriptomic profiles were used to assess the prognostic role of these key genes in renal allograft outcomes after RIRI.

**Results:**

In the bulk-RNA analysis, endoplasmic reticulum stress was identified as the top enriched pathway and three endoplasmic reticulum stress-related genes (PPP1R15A, JUN, and ATF3) were ranked as top performers in both LASSO and Boruta analyses. The three genes were found to significantly interact with kidney injury-related pathways, including apoptosis, inflammatory response, oxidative stress, and pyroptosis. For oxidative stress, these genes were more strongly related to oxidative markers compared with antioxidant markers. In single-cell transcriptome, the three genes were primarily upregulated in endothelium, distal convoluted tubule cells, and collecting duct principal cells among 12 cell types of renal tissues in RIRI. Furthermore, distal convoluted tubule cells and collecting duct principal cells exhibited pro-inflammatory status and the highest pyroptosis levels, suggesting their potential as main effectors of three key genes for mediating RIRI-associated injuries. Importantly, inhibition of these key genes using 4-phenyl butyric acid alleviated functional and histological damage in a mouse RIRI model. Finally, the three genes demonstrated highly prognostic value in predicting graft survival outcomes.

**Conclusion:**

The study identified three key endoplasmic reticulum stress-related genes and demonstrated their prognostic value for graft survival, providing references for individualized clinical prevention and treatment of postoperative complications after renal transplantation.

## 1 Introduction

Renal ischemia-reperfusion injury (RIRI) refers to the pathological changes in the process of tissue re-oxygenation following ischemia (1). In renal transplantation, allograft kidneys inevitably undergo RIRI, causing delayed recovery of allograft function, acute rejection and even loss of allograft (2, 3). Many potential mechanisms of RIRI have been determined, including inflammatory responses, oxidative damage and endothelial dysfunction (4, 5). In detail, the oxidative and inflammatory status induced by ischemia is further exacerbated by the explosive production and dramatic accumulation of reactive oxygen species (ROS) after reperfusion (2, 3). Several techniques and drugs were developed to prevent or alleviate RIRI, including reduction of renal ischemic time, elimination of ROS, inflammation, and ischemic preconditioning (3, 6–8). Although these methods have been applied in clinical practice, the treatment outcomes for RIRI remain limited. Thus, future studies that explored potential mechanisms underlying RIRI are in unmet need for the development of novel strategies to efficiently prevent or mitigate RIRI.

Endoplasmic reticulum (ER) is a vital organelle for maintaining cellular homeostasis and acting a crucial role in protein synthesis, folding and structural maturation (9). Many factors, including hypoxia, oxidative stress, metabolic abnormalities, iron imbalance, calcium ion leakage, and viral infection, impair the ER protein-folding ability, causing the accumulation of unfolded and misfolded proteins. This resulting disorder of ER homeostasis refers to ER stress (ERS) (10–12). ERS has been reported to be involved in various renal diseases, including genetic mutations, acute kidney injury, diabetic nephropathy, and proteinuria (13). However, the underlying mechanisms of ERS in RIRI remain ambiguous. Several studies have identified ER molecular chaperones (BiP/GRP78 and GRP94) and found that unfolded protein response inducers could induce BiP/GRP78 and alleviate RIRI, suggesting the protective role of ERS in RIRI (14–17). On the contrary, intermedin was reported to protect against RIRI by repressing ERS and ERS-related apoptosis, indicating the pathogenic role of ERS in RIRI (18). Although this apparent discrepancy remains undefined, these experimental results are in accordance with the “double-edged sword” hypothesis of ERS. Specifically, the mild-moderate and severe ERS-induced cytoprotective unfolded protein response and pathological apoptotic pathways, respectively. Owing to the contradictory findings of ERS in RIRI, it is crucial to describe in detail the role of ERS in RIRI and identify key ERS-related genes based on large human and mouse datasets, which might serve as therapeutic targets.

In this research, we integrated bulk transcriptomic and single-cell RNA-seq datasets to elucidate the detailed mechanisms underlying ERS in RIRI. In bulk transcriptomic levels, the inducers and downstream targets of ERS were top upregulated in the renal tissue after reperfusion. Then, we enrolled the ERS-related gene sets and identified three key ERS-related genes, which were consistently upregulated among human and mouse datasets during RIRI. The three key ERS-related genes were strongly correlated with pathological pathways participating in RIRI, including NF-kappa B, inflammatory pathways, apoptosis, oxidative stress and pyroptosis. At the single-cell level, altered expressions of three key ERS-related genes before and after reperfusion were primarily observed in endothelium, DCT and CD-PC. Among these three cell types, DCT and CD-PC exhibited the pro-inflammatory status and the highest pyroptosis levels. In addition, the three genes were demonstrated as risk factors for allograft survival, deteriorated graft function and allograft loss. Overall, our results highlighted the crucial role of the three key ERS-related genes in RIRI and provided evidence for potential RIRI treatments targeting these three genes.

## 2 Materials and methods

### 2.1 Bulk RNA data collection and processing

We enrolled three human bulk RNA-seq datasets (GSE43974, GSE90861 and GSE126805) (19–21) comprising a total of 495 renal samples of pre- and post-reperfusion. The mouse bulk RNA-seq dataset (GSE98622) (22) included various post-reperfusion time points and was used for cross-species validation. In addition, three datasets (GSE21374, GSE52694, and GSE58601) (23–25) containing transcriptomic data of renal allografts and graft outcomes were collected for clinical analysis. All datasets were downloaded from the publicly available GEO database, and data acquisition and application were accorded to GEO publication guidelines and data access policies.

The DESeq2 R package and limma R package were utilized for normalization and differential expressed gene (DEG) analysis of bulk RNA-seq data and bulk RNA array data, respectively (26, 27). For enrichment analysis, the Kyoto Encyclopedia of Genes and Genomes (KEGG) and Gene Ontology (GO) pathway analysis were conducted using the DAVID online enrichment tool (https://david.ncifcrf.gov). The single-sample gene set enrichment analysis (ssGSEA) by the gsva R package was implemented with gene sets as the reference, including hallmarks obtained from the Molecular Signatures Database (MSigDB) and cell-death-related gene signatures (28). The protein-protein interaction (PPI) networks were acquired from the STING online database and visualized by the Cytoscape software. The glmnet R package was used to apply the least absolute shrinkage and selection operator (LASSO) regression analysis to select candidate genes for further study. The relevance scores obtained from GeneCards presented the correlations between key genes and oxidative stress markers. The interactions with a relevance score ≥ 5 were applied by the Cytoscape software to construct networks of key genes and oxidative stress markers. The CIBERSORT algorithm (29) was utilized to infer the 22 immune cell populations of renal allografts. For the construction of miRNA- and transcription factors (TFs)-gene regulatory networks, miRNet (https://www.mirnet.ca/) and NetworkAnalyst (https://www.networkanalyst.ca/) were accessed to obtain miRNA and upstream TFs of selected genes, respectively (30, 31). Additionally, the Kaplan–Meier survival curve was used for assessing the prognostic value of key genes using the survival (https://github.com/therneau/survival) and survminer (https://github.com/kassambara/survminer) R packages.

### 2.2 Collection and analysis of single-cell transcriptome data

The mouse single-cell dataset (GSE161201) (32) contained one normal renal sample and two RIRI samples (samples from 6 hours and 24 hours post-reperfusion). The single-cell transcriptome data was processed and integrated using the Seurat R (33) and Harmony R (34) packages, respectively. The single-cell data matrices were filtered by custom criterion (cells expressing 500~3000 and with proportions of mitochondrial genes < 50% and ribosomal genes < 10% were retained). Marker genes for clusters were selected by the Seurat FindAllMarkers function (genes at least detected in 25% of cells in target population cells, log_2_FC > 0.25). Odds ratios (OR) of each cell cluster were calculated and characterized the tissue or sample distribution of meta-clusters (35). Cell–cell interaction analysis was conducted by the CellChat R package (www.cellchat.org). For enrichment analysis of target cell types, the Seurat FindMarkers function was implemented to calculate the log fold change of genes among different groups, applying for the gene set enrichment analysis (GSEA) with hallmarks as reference. The irGSEA R package (https://github.com/chuiqin/irGSEA/) was utilized to calculate the singscore of specific gene sets for single cells. **Supplementary Table 1** summarizes detailed information on the above data sets used in this study.

### 2.3 Experimental mouse RIRI model and estimation of renal damage

In animal experiments, twenty-four C57BL/6 mice (8 weeks old, male) were purchased from Weitonglihua (Beijing, China) and housed under a 12-hour light dark cycle with free access to food and water. All mice were kept in a pathogen-free environment and given a week to adapt to the conditions. The 4-phenyl butyric acid (4-PBA) (100mg/kg, intraperitoneally injected 1 h prior renal ischemia, MCE), an ERS inhibitor, was dissolved in phosphate buffered saline (PBS) and sodium hydroxide was applied to adjust pH to 7.4. Mice were categorized into four groups, including the sham group with PBS (sham + PBS group, n=6), IRI group with PBS (IRI + PBS group, n=6), IRI group with 4-PBA (IRI + 4-PBA group, n=6) and sham group with 4-PBA (sham + 4-PBA group, n=6). RIRI models were conducted with the following procedures: after anesthetized with intraperitoneal injection of pentobarbital, the right kidney was cut, and the left renal pedicle was clamped for thirty minutes in a heating pad (34°C - 36°C), followed by blood reperfusion for 24h. Mice in sham + PBS and sham + 4-PBA groups experienced the same processes without clamping of the left renal pedicle. All mice were euthanized 24 hours after corresponding operation and blood samples and left kidneys were then collected. The renal samples isolated from mice were fixed in 4% paraformaldehyde, embedded in paraffin and stained with Hematoxylin and eosin (H&E), periodic acid-Schiff staining (PAS) and terminal deoxynucleotidyl transferase dUTP nick end labeling (TUNEL) to assess renal histological injury in general. The blood samples were centrifugated at a speed of 3000 revolutions per minute for 10 minutes to get serum samples for blood urea nitrogen (BUN) and creatinine (Cre) measurements by an automated chemistry analyzer (Chemray 800).

### 2.4 qRT-PCR analysis

Total RNA was isolated from the kidneys using TRIzol reagent and then reverse transcribed to cDNA by a cDNA synthesis kit. The mRNA expression levels were normalized to the Ct values of the internal control gene (GAPDH), and fold changes were calculated compared with the control samples. Each sample was performed in triplicate in independent experiments. The primer sequences of three key ERS-related genes were listed in **Supplementary Table 5**.

### 2.5 Statistical analysis

The normality of the variable distribution was assessed using the D’Agostino and Pearson omnibus normality tests. Parameters with normal distribution were conducted contrasts by a two-tailed unpaired t-test and the Pearson correlation analysis. For variables that did not follow a normal distribution, the Mann-Whitney U test and Spearman correlation were employed. A significance level of less than 0.05 was considered statistically significant. We performed R software (version 4.0.5; http://www.r-project.org/) for statistical analysis and graphical representations.

## 3 Results

### 3.1 Biological processes and pathways activated during RIRI

**Figure 1:**
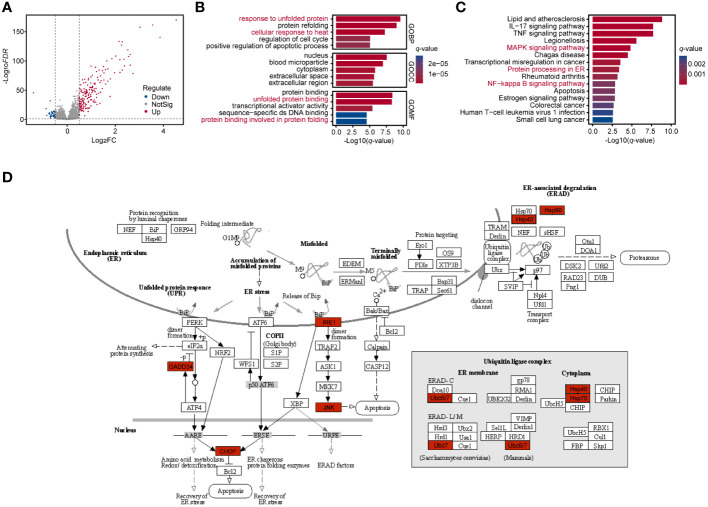
Enrichment analysis of renal tissues during RIRI. **(A)** Volcano plot shows DEGs between post-reperfusion and pre-reperfusion samples. Blue and red dots represent significantly downregulated genes and upregulated genes, respectively. **(B, C)** Bar plots showing top 5 Gene Ontology (GO) and Kyoto Encyclopedia of Genes and Genomes (KEGG) pathways for significantly differentially expressed genes during RIRI. Bar length and color represent log10-transformation with q-value of their corresponding pathways. **(D)** Top disturbed pathway during reperfusion of ischemically injured kidneys, namely Protein processing in ER (modified from KEGG pathway hsa04141). Significantly differentially expressed genes are indicated in red. RIRI, renal ischemia-reperfusion injury; DEG, differential expressed gene; ER, endoplasmic reticulum.

The DEG analysis for GSE43974 was performed and identified 219 DEGs (177 up-regulated genes and 42 down-regulated genes) (**Supplementary Table 2**) based on the criterion (|logFC| > 0.5 and *FDR* < 0.05) (**Figure 1A**). The significantly enriched GO terms based on DEGs referred to “response to unfolded protein”, “response to heat”, “unfolded protein binding” and “protein binding involved in protein folding” (**Figure 1B**; **Supplementary Table 3**). The functionally KEGG pathway analysis displayed that the significantly top pathways involved in RIRI were the MAPK signaling pathway, protein processing in ER and NF−kappa B signaling pathway (**Figure 1C**; **Supplementary Table 3**). Overall, biological GO and KEGG pathway analyses indicated a response of renal tissues to unfolded proteins in the ER during post-reperfusion. Co-activation of the ERS, MAPK pathway and apoptosis as evidenced by significantly upregulated c-Jun N-terminal kinase (JNK) and CHOP (36) during RIRI pointed to interactions between ERS, JNK/p38 MAPK signaling and apoptosis (**Figure 1D**). Additionally, nuclear factor-kB (NF-kappa B) was demonstrated to be the target of ERS (37). In brief, ERS inducers and downstream targets were activated in the renal tissue after reperfusion.

### 3.2 Identification and verification of key ERS-related genes during RIRI

**Figure 2:**
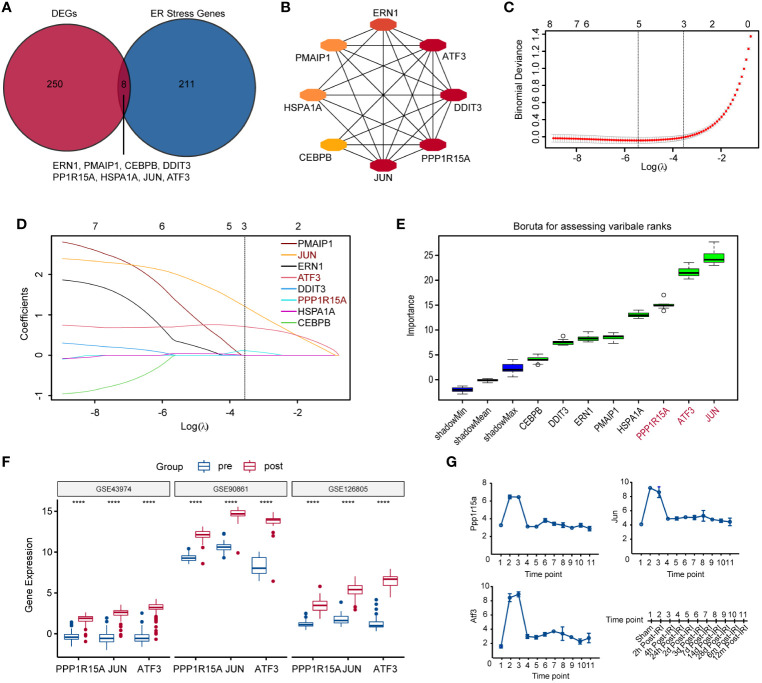
Identification and verification of key ERS-related genes during RIRI. **(A)** Veen plot shows the intersection of differentially significantly expressed genes (DEGs) and ERS-related genes. **(B)** The protein-protein interaction (PPI) network displaying eight tightly intersected genes. **(C)** The coefficient profiles of the LASSO regression model. **(D)** Cross-validation for tuning parameter screening in the LASSO regression model. **(E)** Boruta plot showing the importance (y-axis) of eight intersected genes. **(F)** Box plots showing the expression of three key ERS-gene after reperfusion in GSE43974, GSE90861 and GSE126805. **(G)** Line plots show changes in expression levels of three key ERS-related genes controls and RIRI samples at various post-reperfusion stages, including eleven different time points as illustrated on the bottom-right side. ERS, endoplasmic reticulum stress; LASSO, least absolute shrinkage and selection operator. ****p<0.0001.

ERS-related genes (n=258) from two ERS-related gene sets (including GO RESPONSE TO ENDOPLASMICRETICULUM STRESS and GO REGULATION OF RESPONSE TO ENDOPLASMIC RETICULUM STRESS) were obtained from Molecular Signature Database v7.0 (MSigDB). Then, eight genes (*ATF3, PPP1R15A, CEBPB, PMAIP1, HSPA1A, ERN1, DDIT3*, and *JUN*) were selected through an intersection analysis between ERS-related genes and DEGs (**Figure 2A**). The PPI network showed that these eight genes were all strongly linked (**Figure 2B**). Three key genes (*PPP1R15A*, *JUN* and *ATF3*) were identified from these eight genes using the LASSO algorithm (**Figures 2C, D**), which ranked the top three by Boruta analysis (**Figure 2E**). Then, we found that these three key genes were also significantly elevated in two additional datasets (GSE90861 and GSE126805) during RIRI (**Figure 2F**). The mouse dataset (GSE98622) included transcriptomic data of sham control kidneys and post-reperfusion renal tissues at various post-reperfusion time points (2h, 4h, 24h, 2d, 3d, 7d, 14d, 28d, 6m and 12m). The time course analysis of three key genes using the GSE98622 dataset revealed that gene expression levels were significantly elevated during early reperfusion (2h, 4h and 24h) (**Figure 2G**). In addition, we also found that these three gene levels 6 months after RIRI were higher compared to pre-reperfusion samples (**Figure 2G**), suggesting that these three gene levels might serve as an indicator for the long-term prognosis of patients with renal transplantation.

### 3.3 Three key ERS-related genes exhibited closely related to inflammatory pathways, apoptosis, oxidative stress and pyroptosis during RIRI

**Figure 3:**
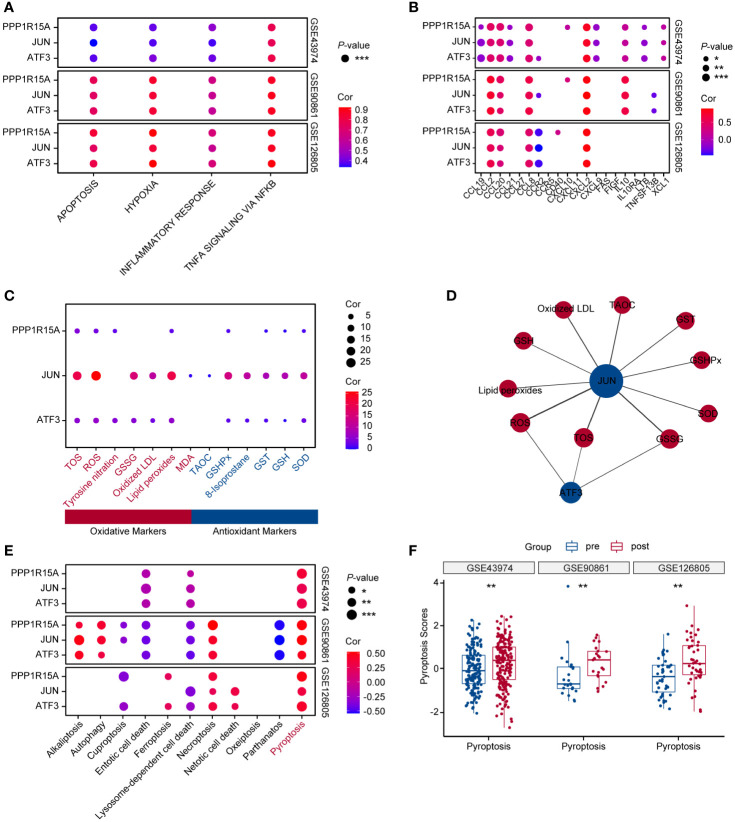
Key ERS-related genes show a crucial role for inflammatory pathways, apoptosis, oxidative stress and pyroptosis during RIRI. **(A)** Bubble chart displaying correlation analysis between key ERS-related gene expression levels and RIRI-related pathway scores. Bubble size and color are represented with p-values and coefficients of correlation analysis in GSE43974, GSE90861 and GSE126805. **(B)** Bubble plot showing correlation analysis between three key gene expression values and inflammatory gene expression levels. The p-values and coefficients of correlation analysis are indicated in bubble size and color. **(C)** Bubble plot of the relevance scores of key ERS-related gene expression levels and oxidative stress. Red text represents oxidative markers, and blue text represents antioxidant markers. **(D)** The networks of key ERS-genes with oxidative markers. Oxidative biomarkers and antioxidant biomarkers are indicated in red and blue. **(E)** Bubble plot of the relevance scores of key ERS-related gene expression levels and 11 programmed cell death levels. **(F)** Box plots showing pyroptosis scores between pre-and post-reperfusion in GSE43974, GSE90861 and GSE126805. *p < 0.05, **p<0.01, ***p<0.001.

In three independent RIRI datasets, the levels of apoptosis, hypoxia, inflammatory response and TNFα signaling via NF-kappa B were significantly higher than in pre-reperfusion samples (**Supplementary Figures S1A–D**). The correlation results showed that three key genes positively correlated with these kidney injury-related pathways across three human RIRI datasets (**Figure 3A**). Furthermore, inflammatory genes (*CCL2, CCL20, CCL8* and *CXCL2*) were demonstrated to positively correlate with all three genes. The chemokine genes (*CCL2, CCL20, CCL8* and *CXCL2*) displayed chemotactic activity for monocytes, basophils, lymphocytes and eosinophils (**Figure 3B**). However, analysis of immune cell abundances based on the bulk RNA dataset indicated that only eosinophils abundances showed significantly increased after reperfusion (**Supplementary Figures S2A–D**). Moreover, eosinophils infiltrating abundances were positively correlated with three key ERS-related gene expression levels (sure S2E). The GeneCard database was applied for correlation analysis between oxidative stress markers and the three genes, revealing that these genes were more strongly related to oxidative markers than antioxidant markers (**Figure 3C**). The networks of the three genes revealed that the oxidative markers, including total oxidant status (TOS), ROS and oxidized glutathione (GSSG) were strongly correlated with both *JUN* and *ATF3* (**Figure 3D**). In addition, among 11 PCD-related gene sets (28), pyroptosis was strongly and positively correlated with key ERS-gene expression levels across three human datasets (**Figure 3E**). The pyroptosis scores after RIRI exhibited significantly higher than those of pre-reperfusion in the three human datasets (**Figure 3F**).

### 3.4 Prediction of miRNA and TFs for three key ERS-related genes

The miRNA- and TF-mRNA interaction networks were comprised of 137 miRNAs and 174 TFs, respectively. For miRNA-mRNA regulatory interactions, 31 miRNAs, 113 miRNAs and 31 miRNAs target *PPP1R15A, JUN* and *ATF3*, respectively (**Supplementary Figure S3A**). Among them, ten miRNAs (hsa-mir-1-3p, hsa-mir-10b-5p, hsa-mir-16-5p, hsa-mir-17-5p, hsa-mir-21-3p, hsa-mir-24-3p, hsa-mir-30a-5p, hsa-mir-34a-5p, hsa-mir-124-3p and hsa-mir-191-5p) could regulate all three key ERS-related genes (**Supplementary Figure S3A**). For TF-mRNA regulatory interactions, *PPP1R15A, JUN* and *ATF3* could be regulated by 120 TFs, 73 TFs and 39 TFs, respectively (**Supplementary Figure S3B**). Eight TFs, including *ZFP37, SMAD5, REST, RAD21, KLF16, FOXJ2, ELF1*, and *BCL11B*, could regulate all three key ERS-related genes (**Supplementary Figure S3B**).

### 3.5 Altered expressions of key ERS-related genes during RIRI injury were primarily in the endothelium, DCT and CD-PC

**Figure 4:**
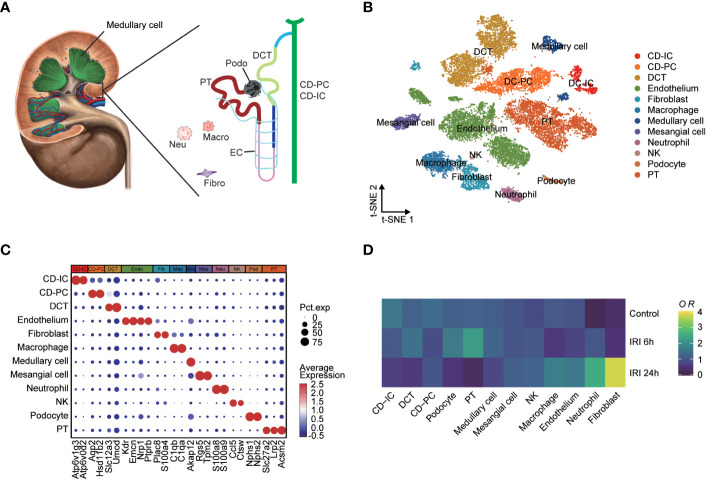
Single-cell characterization of mouse IRI kidneys. **(A)** Main cell types of mouse human kidney (CD-IC, collecting duct intercalated cells; CD-PC, collecting duct principal cells; DCT, distal convoluted tubule; EC, endothelium; Fibro, fibroblasts; Macro, macrophages; Neu, neutrophils; Podo, podocytes; PT, proximal tubule). **(B)** tSNE plot showing 16,658 cells from the sham control, IRI-6h and IRI-24h samples, colored by 12 major cell types. **(C)** Dotplot displaying the percent expressed cells and average expression levels of marker genes of 12 cell lineages. **(D)** Heatmap of relative abundances for each cell types among the sham control, IRI-6h and IRI-24h groups. The OR levels represent the cell abundances and range from 0 (blue) to 4 (yellow). tSNE, t-distributed stochastic neighbor embedding; OR, odds ratios.

**Figure 5:**
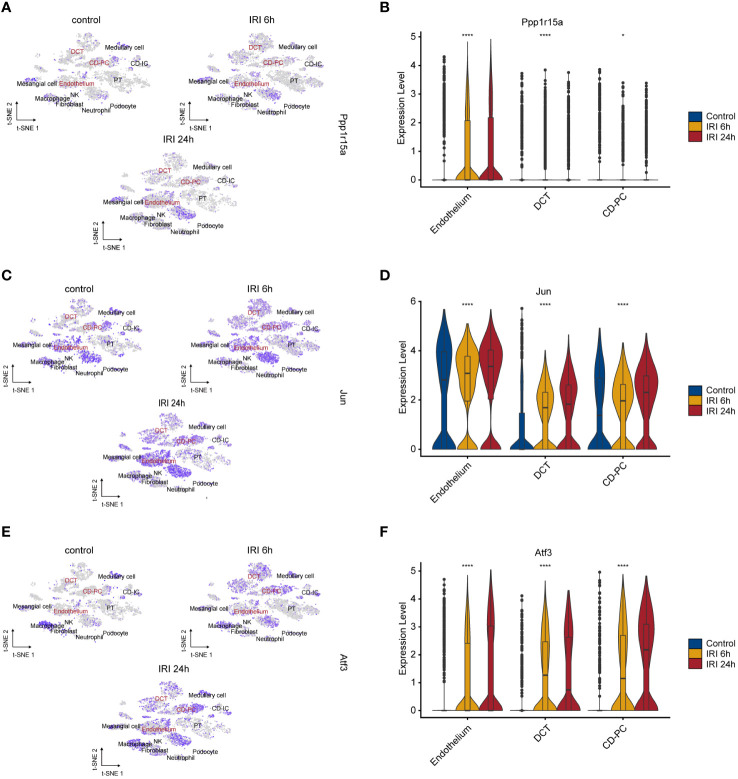
Characteristics of key ERS-gene expression levels during renal IRI. tSNE plots showing the expression of PPP1R15A **(A)**, JUN **(C)** and ATF3 **(E)** for 12 cell types. Intensities of color reveal normalized gene expression. Violine plots depicting significantly upregulation of PPP1R15A **(B)**, JUN **(D)** and ATF3 **(F)** in 6h and 24h post-reperfusion samples compared with sham control sample. *p<0.05, ****p<0.0001.

The single-cell RNA-seq dataset (GSE161201) included 22,310 cells, comprising 5,246 cells from the sham control sample, 10,393 cells from the RIRI-6h sample and 6,671 cells from the RIRI-24h sample (**Supplementary Figure S4A**). After filtration, 16,658 cells were obtained, including 4,282 cells from the sham control sample, 6,515 cells from the RIRI-6h sample, and 5,861 cells from the RIRI-24h sample. Next, a total of 22 clusters within the integrated dataset were identified and visualized by the t-SNE algorithm (**Supplementary Figure S4B**). The anatomical mapping of various cell types to specific nephron segments were presented due to the complexity of kidney tissue (**Figure 4A**). The clusters were combined and assigned to 12 known cell types based on the marker genes from the previous studies (38–40) and CellMarker database (http://xteam.xbio.top/CellMarker/) (**Figures 4B, C**; **Supplementary Table 4**). The OR of each cell type was calculated and visualized by the heatmap to characterize the distribution of the 12 known cell lineages among the three groups. The ORs of cell types showed that renal tissues of 24h post-reperfusion exhibited higher levels offibroblasts, neutrophil cells and macrophages than renal tissues of the control and 6h post-reperfusion (**Figure 4D**). Cell-cell interaction analysis showed that the number of interactions and interaction strengths between neutrophils and other cell types were elevated after reperfusion (**Supplementary Figures S4C, D**). The gene expression density maps were analyzed to explore cell-specific changes in key ERS-related gene expression levels for each sample. The key ERS-related genes were mainly upregulated in endothelium, CD-PC and DCT of RIRI-6h or RIRI-24h samples compared with the sham control sample (**Figures 5A–F**). These results supported weak connections between the key ERS-gene levels and most immune cell abundances by bulk RNA-seq analysis, suggesting the vital role of ERS on non-immune cells (endothelium, CD-PC and DCT) during RIRI damage.

### 3.6 DCT and CD-PC presented with pro-inflammatory status and high pyroptosis scores

**Figure 6:**
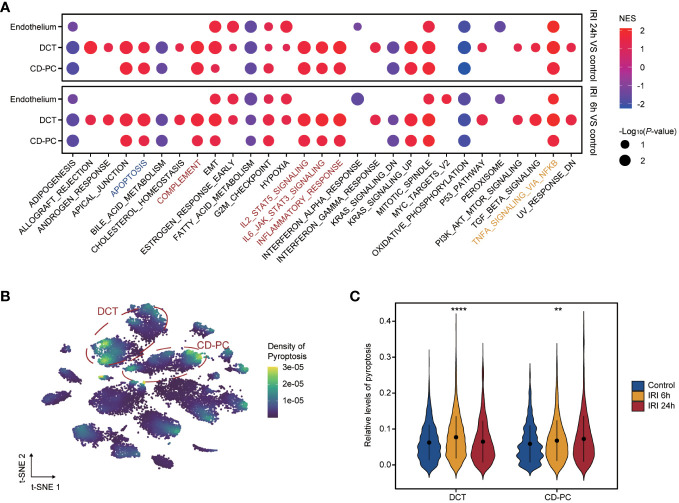
Functional profile of endothelium, CD-PC and DCT during renal IRI. **(A)** Dotplot showing the enriched hallmarks of endothelium, CD-PC and DCT between IRI 6h versus control versus e or IRI 24h versus control. Dot color and size present normalized enrichment scores (NES) and p-values obtained from GSEA analysis. The apoptosis, inflammatory pathways and TNFα signaling via NF-kappa B are labelled with blue, red and yellow colors. **(B)** tSNE plot revealing pyroptosis densities of 12 cell types. DCT and CD-PC cells circled in red exhibit the top density of pyroptosis. **(C)** Violine plot displaying significantly elevated pyroptosis levels in IRI 6h and IRI 24h samples compared with control sample in DCT and CD-PC. CD-PC, collecting duct principal cells; DCT, distal convoluted tubule; GSEA, gene set enrichment analysis. **p<0.01, ****p<0.0001.

Functional enrichment analysis of the endothelium, CD-PC and DCT in RIRI-6h or RIRI-24h samples compared with the control sample showed a consistently significant enrichment of TNFα signaling via NF-kappa B (**Figure 6A**). Apart from the endothelium, DCT and CD-PC exhibited enrichment of pro-inflammatory pathways (complement, inflammatory response, IL-2/STAT5 signaling and IL-6/JAK/STAT3 signaling pathways) and apoptosis (**Figure 6A**). Based on these results, we speculated that DCT and CD-PC with pro-inflammatory and apoptotic status served as main senders or targets of ERS for mediating RIRI injury. In addition, density maps were applied to estimate the relevance scores of pyroptosis for each cell type and showed that DCT and CD-PC exhibited the highest pyroptosis scores among all cell types examined (**Figure 6B**). In both DCT and CD-PC, the relative levels of pyroptosis were significantly increased in the RIRI-6h or RIRI-24h samples compared with the sham control sample (**Figure 6C**). In brief, pyroptosis responses were mainly in DCT and CD-PC among other renal cells and were significantly elevated during RIRI.

### 3.7 Inhibition of ERS alleviated RIRI and suppressed three key ERS-related genes in mice

**Figure 7:**
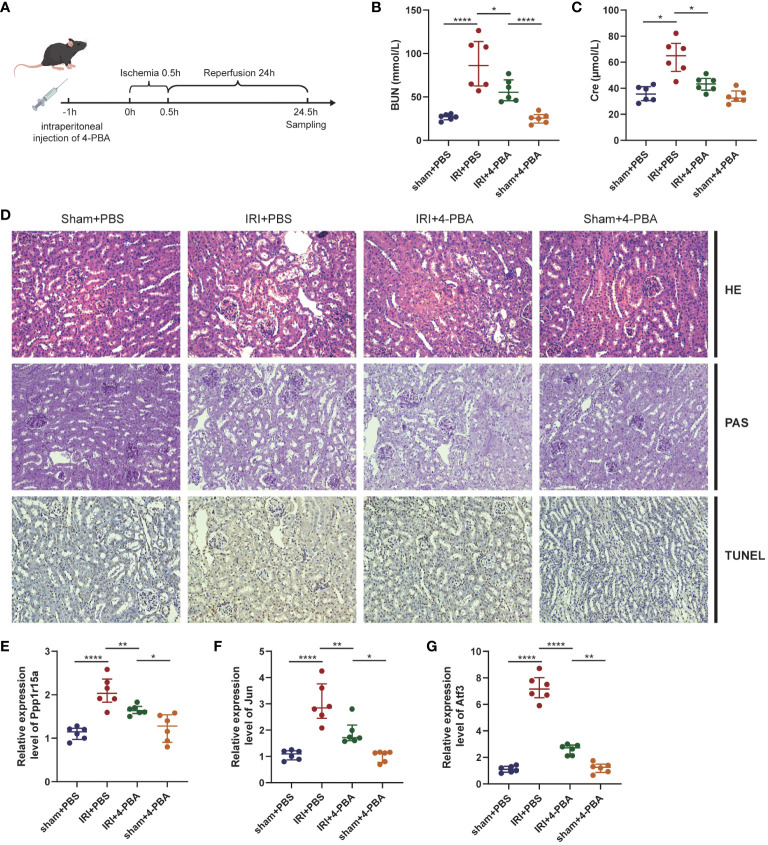
ERS in RIRI mouse model. **(A)** Pharmacotherapy of RIRI mouse model. The 4-phenyl butyric acid (4-PBA) is administered intraperitoneally to the mice 1 hour before ischemia. **(B, C)** Serum BUN and blood urea nitrogen (BUN) and creatinine (Cre) levels in mice. **(D)** Representative images of HE, PAS and TUNEL staining (×200 magnification) of renal tissues. mRNA levels of key ERS-related genes [PPP1R15A **(E)**, JUN **(F)** and ATF3 **(G)**] in RIRI mouse model. *P < 0.05, **P < 0.01, ****P < 0.0001, HE: hematoxylin and eosin staining, PAS, periodic acid-Schiff staining, TUNEL, terminal deoxynucleotidyl transferase dUTP nick end labeling staining.

A mouse RIRI model with or without 4-PBA was constructed to further unveil the role of ERS in RIRI (**Figure 7A**). The results showed that BUN and Cre were significantly elevated after reperfusion and renal impairment induced by RIRI was greatly ameliorated by 4-PBA application, manifested as the amelioration of functional damage (**Figures 7B, C**) and histological injuries (**Figure 7D**). Thus, we concluded that inhibition of ERS improved renal ischemia/reperfusion-associated injuries. The expression levels of three key ERS-related genes were further verified by qRT-PCR analysis. Compared with the sham group, the expression levels of these genes after RIRI were significantly higher than those in sham samples and greatly decreased in the group treated with 4-PBA (**Figures 7E–G**).

### 3.8 Prognostic value evaluation of key ERS-related genes

**Figure 8:**
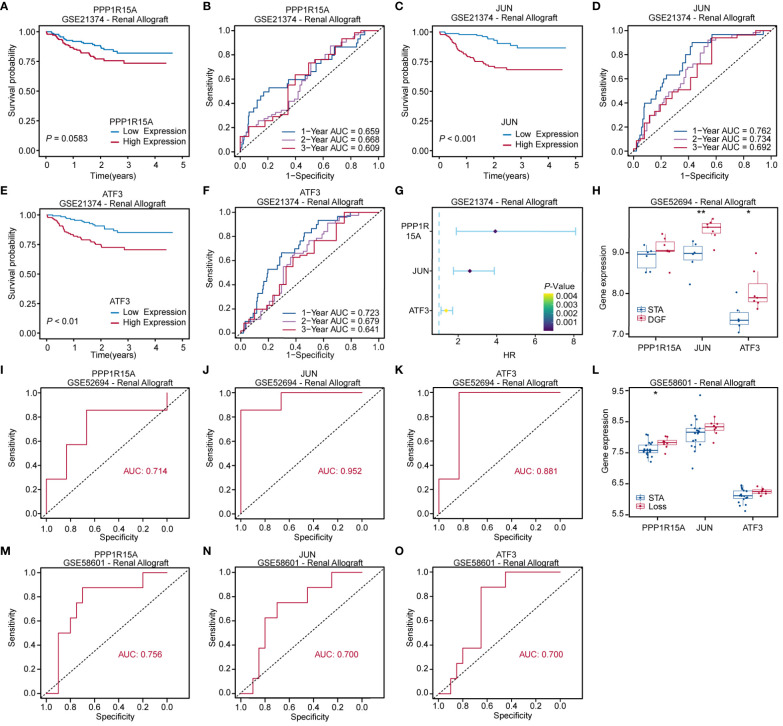
Prognostic value evaluation of key ERS-related genes. Kaplan-Meier and receiver operating characteristic (ROC) curves assessing prognostic values of PPP1R15A **(A, B)**, JUN **(C, D)** and ATF3 **(E, F)** expression levels for kidney recipients. **(G)** Forest plot based on univariable Cox regression analysis showing that key ERS-related genes are significantly risk factors for renal allograft survivals. **(H)** Boxplot comparing key ERS-related gene expression levels between kidney recipients with stable renal function (STA) and deteriorated graft function (DGF). **(I–K)** ROC curves showing that key ERS-related genes were able to accurately predict renal function after renal transplantation. **(L)** Boxplot comparing key ERS-related gene expression levels between kidney recipients with STA and allograft loss. **(M–O)** ROC curves revealing that three key genes exhibited acceptable predictive performance in renal allograft outcomes. *p<0.05, **p<0.01.

**Figure 9:**
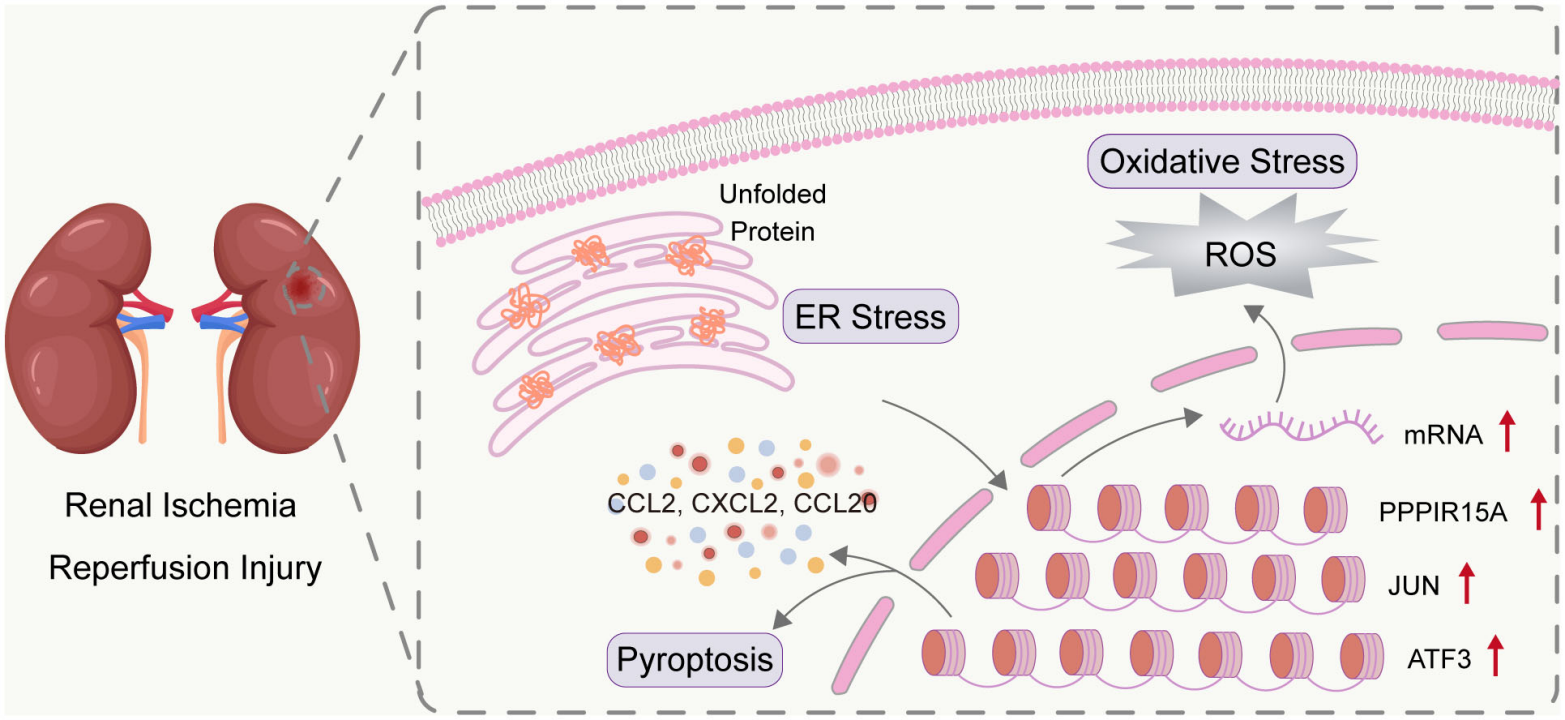
Overview of three key ERS-related genes in RIRI. RIRI induced the accumulation of unfold proteins and thus mediating the activation of ERS. The three genes (*PPP1R15A*, *JUN*, and *ATF3*) were determined as crucial ERS genes involved in RIRI. The three genes were biologically related to kidney-injury pathways (including inflammation, oxidative stress, and pyroptosis) and had clinical value in both acute injury and late graft outcomes.

The 282 kidney recipients in the GSE21374 dataset were divided into high and low gene-expression groups dichotomized at the corresponding median of each key ERS-related gene level. Kaplan-Meier survival curves showed a trend toward worse prognosis for recipients with high expression of *PPP1R15A, JUN* and *ATF3* compared to those with low expression (**Figures 8A, C, E**). The area under the curve (AUC) of predictions for 1, 2, and 3 years was depicted in **Figures 8B, D, F**. The highest AUC values of *PPP1R15A, JUN* and *ATF3* were 0.668, 0.762, and 0.723, respectively. Besides, all three key ERS-related genes were significant risk factors for renal allograft survival (**Figure 8G**). The GSE52694 dataset included RNA profiles of 13 renal graft samples from patients with the diagnosis of borderline changes early (≤2 months). These samples were categorized into stable (STA, n=6) and deteriorated graft function (DGF, n=7) groups during 2 years after renal transplantation. All key ERS-related genes exhibited an increasing trend in the DGF group and JUN and ATF3 were significantly enhanced in the DGF group (**Figure 8H**). The receiver operating characteristic (ROC) curves indicated that all three key ERS-related genes with a high degree of reliability in accurately predicting allograft outcomes (PPP1R15A: AUC = 0.714; JUN: AUC = 0.952; ATF3: AUC = 0.881) (**Figures 8I–K**). In addition, a total of 28 renal biopsy samples (STA, n=20; graft loss, n=8) were applied for gene expression profiling and it was found that all three genes showed higher levels in the graft loss group than those in the STA group (**Figure 8L**). The AUCs of three key genes in predicting renal graft outcomes were greater than 0.7, which was considered as an acceptable predictive accuracy (**Figures 8M–O**). Overall, the three key ERS-related genes have prognostic values in predicting graft survival outcomes and assessing the risk of DGF and graft loss and the primary mechanisms by which the three genes affect RIRI are depicted schematically in **Figure 9**.

## 4 Discussion

RIRI, a common tissue after renal transplantation, causes delayed graft function, increases the risk of renal allograft rejection and even contributes to graft loss (2, 3). However, the underlying mechanisms of RIRI are complicated and there is no FDA-approved drugs for the treatment of RIRI in clinics (41). To fully determine the mechanisms of RIRI and identify potential new targets, we collected all available RIRI datasets with a large sample size and performed bulk RNA-seq analysis on these samples. Results showed that ERS was the top enriched pathway in RIRI. Among ERS-related genes, three key genes (*PPP1R15A*, *JUN* and *ATF3*) ranked top based on machine learning algorithms and were found to positively correlate with kidney injury-related pathways, including apoptosis, inflammatory response, oxidative stress, and pyroptosis. Our single-cell RNA-seq analysis suggested that DCT and CD-PC with pro-inflammatory status and the highest pyroptosis levels in RIRI were the main effectors of the three key genes. Furthermore, inhibition of the three key genes alleviated the functional and histological damage of RIRI. Importantly, renal allograft recipients with high levels of the three genes exhibited poor prognosis in long-term outcome and graft survival. The study is the first analysis to integrate multi-omics and clinical data for determining the crucial roles of three key ERS-related genes in RIRI, providing new ideas for clinical treatment.

Previous reports have shown that ERS is closely related to kidney diseases, including acute kidney injury, diabetic nephropathy and renal fibrosis (13). Hypoxia and ischemia as ER stressors caused the accumulation of misfolded proteins and eventually resulted in ERS (42). Several studies have found that RIRI can induce ERS in renal cells (43, 44). Some studies focusing on ER molecular chaperones identified the protective role of ERS in RIRI, while others on intermedin suggested the pathogenic role of ERS. However, the exact cause of the discrepancy and mechanisms underlying ERS in RIRI are still unclear. Currently, there is a lack of focus on measuring determinants of ERS or key ERS-related genes based on renal tissues of patients with RIRI. Our results showed that ERS was the top enriched pathway during RIRI across 336 samples, manifested as upregulation of ERS inducers and downstream targets, such as IRE1, CHOP, and JNK. For ERS-related genes, three key genes (PPP1R15A, JUN and ATF3) ranked top using machine learning algorithms and were significantly up-regulated in all three bulk-RNA datasets (including 495 samples). Importantly, these independent datasets were obtained from different centers and hence have high clinical heterogeneity. All the aforementioned results indicated the crucial role of ERS and identified three new ERS-related genes for further mechanistic studies of RIRI.

Previous studies have indicated that the three genes are involved in the process of ischemia-reperfusion injury or ischemia. The PERK and IRE pathways were activated in sustained ERS during neonatal hypoxia-ischemia, leading to a transient phosphorylation of eIF2α and an increased induction of PPP1R15A (45). JUN is a member of the AP-1 (Activator Protein-1) transcription factor family and is an important transcription factor downstream of ERK 1/2 and JNK in the signaling cascade, which were activated in response to ERS induced by coronary microembolization, ultimately leading to cardiomyocyte apoptosis (46–48). ATF3 is a gene that encodes a transcription factor, which is upregulated in ischemia-reperfusion injury including brain (49), liver (50), and cardiac microvascular (51). Studies have shown that the heterodimer of ATF3 can induce heat shock protein 27, which activates the Akt pathway and inhibits MEKK1-JNK, thereby inhibiting neuronal apoptosis (49). In addition to brain, liver and cardiac IRI, limited studies have been conducted on elucidating the molecular roles of the three genes in RIRI. Thus, we incorporated correlation analysis to explore significantly enriched pathways associated with the three genes in RIRI. Results revealed that the three genes were correlated with inflammatory response, oxidative stress, NF-kappa B pathway, apoptosis and pyroptosis. ERS can directly initiate the inflammatory pathway, and the activation of the inflammatory pathway releases a large number of inflammatory factors, which in turn triggers ERS. This induces a pro-inflammatory positive feedback loop that further amplifies the inflammatory response (52, 53). In addition to inflammatory responses, ROS have been shown to act a crucial part in the pathology of IRI (54). Oxidative stress produced at the reperfusion stage might induce injury to the insulted tissues. This process is part of the term “oxygen paradox”, in which re-oxygenation of ischemic tissues generates injuries that largely exceed the injuries caused by ischemia alone (55). In recent years, studies showed that ROS destroyed ER functions and initiated unfold protein response and ERS *in vivo* and *in vitro* (56, 57). For NF-kappa B pathway, several studies have shown that ERS can activate the NF-kappa B pathway through facilitating TNF-α expressions and phosphorylation of p38 MAPK/NFκB signaling proteins (58–60). Apart from the NF-kappa B pathway, apoptotic pathways induced by ERS are an important type of apoptosis (61). The activation of the unfolded protein response initiated apoptotic cell death via up-regulation of CHOP, a pivotal marker of ERS (61). Besides, p38 can activate the transcription of CHOP through ATF6 and inhibition of p38 by SB203580 also restrained the expression of ERS markers, supporting the role of MAPK pathway in response to ERS-induced apoptosis. The first study on pyroptosis and RIRI can be traced back to 2014 and demonstrated that RIRI can induce pyroptosis in renal tubule epithelial cells by the CHOP-caspase-11 pathway (62). Furthermore, several subsequent studies indicated that GSDMD as the protein effector of pyroptosis acted as an executor and contributor to renal I/R injury (63–66). Overall, our results suggested that the three genes were involved in multiple pathological pathways of RIRI rather than a single process, emphasizing their importance. However, the specific mechanisms and the potential upstream and downstream relationships between the three genes and these interrelated pathways still need to be further studied.

Owing to the vulnerability of the renal proximal tubules, most studies have explored the potential mechanisms underlying RIRI using human renal proximal tubule epithelial cell line (HK2) rather than distal tubule cells. Surprisingly, the single-cell RNA-seq analysis indicated that endothelium, DCT and CD-PC exhibited significantly elevated expression levels of the three genes after renal reperfusion. Yoshio et al. also determined that oxygen-regulated protein (ORP150), a key chaperone for ERS in response to RIRI, was principally expressed in distal tubules (67). Besides, we leveraged scRNA-seq of RIRI to show that DCT and CD-PC exhibited pro-inflammatory status and the highest pyroptosis density among all cell types examined. While previous studies have provided a general understanding of the role of pyroptosis in the renal tubular epithelium, few studies have specifically addressed which segment of the tubular is affected (68, 69). We innovatively revealed that DCT and CD-PC may also be vital targets of pyroptosis in RIRI, providing new insights into the mechanism of RIRI. In addition to non-immune cells, innate and adaptive immune cells are also involved in IRI. IRI is a type of sterile inflammatory response but has similarities with the inflammation by pathogens (70). Infiltration of neutrophils occurs as early as 30 minutes after reperfusion, causing interstitial edema activation of the endothelium in peritubular capillaries (71). The neutrophils exacerbate kidney injury through secreting ROS (72) and inflammatory cytokines. Depleting neutrophils by anti ICAM-1 antibody can protect against RIRI (73, 74), demonstrating the determinants of neutrophils in acute kidney injury in mice. However, the pathological role of neutrophils is still not fully validated, and ICAM-1 blocking exhibits no beneficial effect on DGF after RIRI (75). Consistent with these human studies, our study found that the infiltrating neutrophils showed no significant differences between pre- and post-reperfusion in three cohorts. In contrast with bulk RNA-seq analysis, results of single-cell analysis revealed that the numbers of interactions and interaction strengths between neutrophils and other cell types were elevated after reperfusion, suggesting that cell-cell interaction between neutrophils and other cells may be more crucial than the levels of infiltrating neutrophils in RIRI.

The mice model of RIRI successfully validated the pathological role of three key ERS-related genes on acute kidney injury. In detail, compared with the RIRI group with PBS, the RIRI group treated with 4-PBA exhibited lower levels of the three genes and acute renal damage after reperfusion. In addition to acute kidney injury after RIRI, the patients recovered from RIRI may suffer from chronic kidney disease later (76–78). The maladaptive repair is determined to be a vital mechanism for renal fibrosis, inducing RIRI to chronic kidney disease (79). The ERS-induced apoptosis displayed a role in the progression of kidney fibrosis in the rat model (80). Thus, we collected three datasets with long-term follow-ups and found that the three genes exhibited highly prognostic values in renal allograft outcomes. Taken together, the three genes have clinical value for both acute injury and late graft outcomes. The 4-PBA has notable safety profiles *in vivo*, which is approved by the U.S. Food and Drug Administration for clinical use in urea-cycle disorders (81–83), suggesting its potential in RIRI treatment. However, the 4-PBA inhibited a panel of ERS-related genes beyond just the three genes, and the impact of 4-PBA on the late development of renal fibrosis still needed to be evaluated. Although important roles for ERS were recognized by multi-omics analysis, only a small portion of ERS-relate genes were differentially expressed during RIRI. Compared with 4-PBA, new drugs targeting these three genes may result in better efficacy and minimal off-target effects.

The molecules identified in gene-miRNA and gene-TF regulatory networks may offer some clues to better understand the mechanisms of RIRI and can be potential drug targets. MiRNAs as small RNAs (18-24 nt in size) regulate the post-transcription of mRNAs and hence are involved in multiple biological processes, such as cell survival and stress response. Results showed that ten miRNAs (including hsa-mir-34a-5p, hsa-mir-30a-5p, hsa-mir-24-3p, hsa-mir-21-3p, hsa-mir-191-5p, hsa-mir-17-5p, hsa-mir-16-5p, hsa-mir-1-3p, hsa-mir-124-3p and hsa-mir-10b-5p) interacted with all three key ERS-related genes. Unsurprisingly, nine miRNAs of these ten miRNAs have been demonstrated to independently predict the occurrence of RIRI or prevent the development of RIRI in previous studies (84–91). The miRNAs, including miR-10b-5p, miR-16-5p, miR-24-3p, miR-34a-5p, and miR-191-5p, have been identified to aggravate RIRI through various mechanisms, such as downregulation of PIK3CA expression, regulation of autophagy and promotion of apoptosis. Conversely, miR-21-3p, miR-17-5p, miR-30a-5p, and miR-124-3p have been shown to alleviate IRI by targeting different pathways including caspase signaling pathway, death receptor 6, Beclin 1/ATG16 pathway, and TNFα/RIP1/RIP3 pathway. These findings provide important insights into the role of miRNAs in RIRI and could serve as potential therapeutic targets for future clinical interventions. In addition, eight upstream TFs (including ZFP37, SMAD5, REST, RAD21, KLF16, FOXJ2, ELF1 and BCL11B) could regulate all three key ERS-related genes in RIRI. Among these eight TFs, only REST has been confirmed to involve the process of RIRI. The previous study found that REST was a crucial inducer of ferroptosis in RIRI and inhibiting REST can alleviate the progression of chronic kidney disease from RIRI (92). The results of these studies suggest a great potential for these regulatory miRNAs and TFs as a class of therapeutic targets. The development of drugs may achieve better for controlling better through incorporating these regulatory molecules and ERS-related genes rather than focusing solely on ERS-related genes.

Our study still had some limitations. First, the living donor (LD) to deceased donor (DD) ratio of bulk-RNA datasets (GSE43974, GSE90861 and GSE126805) selected in the current research are different and the biological differences will inevitably produce an impact on our findings. Second, the RIRI mouse model has inherent limitations in recapitulating RIRI in human kidney transplantation (93, 94). Apart from their species heterogeneity, transplant process-related factors including organ retrieval, preservation, transport, and implantation can introduce additional variables and potential sources of injuries that are not present in the controlled laboratory environment of a mouse study (95, 96). Despite these limitations, the use of mouse models provides valuable mechanistic insights and serves as an initial screening platform for studying RIRI. Previous studies have demonstrated that there is a significant overlap between gene sets associated with IRI in both mouse models and published microarray data from deceased donor transplants of human kidneys (19, 22). Finally, additional clinical sample data needs to be established for further verifying the expression levels of the three genes and their correlations with clinical parameters.

## 5 Conclusion

In summary, we performed a multi-omics analysis to identify three key ERS-related genes in RIRI and identified their related biological processes involved in kidney injuries. The three genes have clinical value in both acute injury and late graft outcomes. The 4-PBA inhibiting the three genes alleviated acute kidney injury after RIRI, and its therapeutic impact on long-term outcomes is needed to be explored. In addition, there is an urgent need for new drugs specifically targeting the three genes, which may result in better efficacy and minimal off-target effects.

## Data availability statement

The original contributions presented in the study are included in the article/**Supplementary Material**. Further inquiries can be directed to the corresponding authors.

## Ethics statement

The animal study was reviewed and approved by the ethics committee of Beijing Chaoyang Hospital. The study was conducted in accordance with the local legislation and institutional requirements.

## Author contributions

HZ: Conceptualization, Methodology, Visualization, Writing – original draft. CZ: Methodology, Visualization, Validation, Writing – original draft. YX: Conceptualization, Supervision, Methodology, Writing – review & editing. XH: Conceptualization, Funding acquisition, Supervision, Writing – review & editing.

## References

[1] Zhao H Alam A Soo AP George AJT Ma D. Ischemia-reperfusion injury reduces long term renal graft survival: mechanism and beyond. EBioMedicine. (2018) 28:31–42. doi: 10.1016/j.ebiom.2018.01.025 29398595 PMC5835570

[2] Zeng M Wei X Wu Z Li W Li B Fei Y. Reactive oxygen species contribute to simulated ischemia/reperfusion-induced autophagic cell death in human umbilical vein endothelial cells. Med Sci Monit. (2014) 20:1017–23. doi: 10.12659/MSM.890897 PMC4074109 24943908

[3] Lutz J Thürmel K Heemann U. Anti-inflammatory treatment strategies for ischemia/reperfusion injury in transplantation. J Inflamm (Lond). (2010) 7:27. doi: 10.1186/1476-9255-7-27 20509932 PMC2894818

[4] Fang DYP Lu B Hayward S de Kretser DM Cowan PJ Dwyer KM. The role of activin A and B and the benefit of Follistatin treatment in renal ischemia-reperfusion injury in mice. Transplant Direct. (2016) 2:e87. doi: 10.1097/TXD.0000000000000601 27830181 PMC5087569

[5] Chouchani ET Pell VR James AM Work LM Saeb-Parsy K Frezza C. A unifying mechanism for mitochondrial superoxide production during ischemia-reperfusion injury. Cell Metab. (2016) 23:254–63. doi: 10.1016/j.cmet.2015.12.009 26777689

[6] Tennankore KK Kim SJ Alwayn IPJ Kiberd BA. Prolonged warm ischemia time is associated with graft failure and mortality after kidney transplantation. Kidney Int. (2016) 89:648–58. doi: 10.1016/j.kint.2015.09.002 26880458

[7] Nakagawa K Koo DDH Davies DR Gray DWR McLaren AJ Welsh KI. Lecithinized superoxide dismutase reduces cold ischemia-induced chronic allograft dysfunction. Kidney Int. (2002) 61:1160–9. doi: 10.1046/j.1523-1755.2002.00217.x 11849471

[8] Hwang JK Kim JM Kim YK Kim SD Park SC Kim JI. The early protective effect of glutamine pretreatment and ischemia preconditioning in renal ischemia-reperfusion injury of rat. Transplant Proc. (2013) 45:3203–8. doi: 10.1016/j.transproceed.2013.08.028 24182785

[9] Oakes SA Papa FR. The role of endoplasmic reticulum stress in human pathology. Annu Rev Pathol. (2015) 10:173–94. doi: 10.1146/annurev-pathol-012513-104649 PMC5568783 25387057

[10] So J-S. Roles of endoplasmic reticulum stress in immune responses. Mol Cells. (2018) 41:705–16. doi: 10.14348/molcells.2018.0241 PMC6125421 30078231

[11] Martins AS Alves I Helguero L Domingues MR Neves BM. The unfolded protein response in homeostasis and modulation of mammalian immune cells. Int Rev Immunol. (2016) 35:457–76. doi: 10.3109/08830185.2015.1110151 27119724

[12] Marciniak SJ Ron D. Endoplasmic reticulum stress signaling in disease. Physiol Rev. (2006) 86:1133–49. doi: 10.1152/physrev.00015.2006 17015486

[13] Taniguchi M Yoshida H. Endoplasmic reticulum stress in kidney function and disease. Curr Opin Nephrol Hypertens. (2015) 24:345–50. doi: 10.1097/MNH.0000000000000141 26050121

[14] Bush KT George SK Zhang PL Nigam SK. Pretreatment with inducers of ER molecular chaperones protects epithelial cells subjected to ATP depletion. Am J Physiol. (1999) 277:F211–218. doi: 10.1152/ajprenal.1999.277.2.F211 10444575

[15] Prachasilchai W Sonoda H Yokota-Ikeda N Ito K Kudo T Imaizumi K. The protective effect of a newly developed molecular chaperone-inducer against mouse ischemic acute kidney injury. J Pharmacol Sci. (2009) 109:311–4. doi: 10.1254/jphs.08272SC 19179808

[16] Gao X Fu L Xiao M Xu C Sun L Zhang T. The nephroprotective effect of tauroursodeoxycholic acid on ischaemia/reperfusion-induced acute kidney injury by inhibiting endoplasmic reticulum stress. Basic Clin Pharmacol Toxicol. (2012) 111:14–23. doi: 10.1111/j.1742-7843.2011.00854.x 22212133

[17] Gupta S Li S Abedin MJ Noppakun K Wang L Kaur T. Prevention of acute kidney injury by tauroursodeoxycholic acid in rat and cell culture models. PLoS One. (2012) 7:e48950. doi: 10.1371/journal.pone.0048950 23152827 PMC3494686

[18] Wang Y Tian J Qiao X Su X Mi Y Zhang R. Intermedin protects against renal ischemia-reperfusion injury by inhibiting endoplasmic reticulum stress. BMC Nephrol. (2015) 16:169. doi: 10.1186/s12882-015-0157-7 26498843 PMC4619099

[19] Damman J Bloks VW Daha MR van der Most PJ Sanjabi B van der Vlies P. Hypoxia and complement-and-coagulation pathways in the deceased organ donor as the major target for intervention to improve renal allograft outcome. Transplantation. (2015) 99:1293–300. doi: 10.1097/TP.0000000000000500 25427168

[20] McGuinness D Mohammed S Monaghan L Wilson PA Kingsmore DB Shapter O. A molecular signature for delayed graft function. Aging Cell. (2018) 17:e12825. doi: 10.1111/acel.12825 30094915 PMC6156499

[21] Cippà PE Sun B Liu J Chen L Naesens M McMahon AP. Transcriptional trajectories of human kidney injury progression. JCI Insight. (2018) 3:e123151. doi: 10.1172/jci.insight.123151 30429361 PMC6302941

[22] Liu J Kumar S Dolzhenko E Alvarado GF Guo J Lu C. Molecular characterization of the transition from acute to chronic kidney injury following ischemia/reperfusion. JCI Insight. (2017) 2:e94716. doi: 10.1172/jci.insight.94716 28931758 PMC5612583

[23] Einecke G Reeve J Sis B Mengel M Hidalgo L Famulski KS. A molecular classifier for predicting future graft loss in late kidney transplant biopsies. J Clin Invest. (2010) 120:1862–72. doi: 10.1172/JCI41789 PMC2877953 20501945

[24] Hrubá P Brabcová I Gueler F Krejčík Z Stránecký V Svobodová E. Molecular diagnostics identifies risks for graft dysfunction despite borderline histologic changes. Kidney Int. (2015) 88:785–95. doi: 10.1038/ki.2015.211 26176825

[25] Kamal L Broin PÓ Bao Y Ajaimy M Lubetzky M Gupta A. Clinical, histological, and molecular markers associated with allograft loss in transplant glomerulopathy patients. Transplantation. (2015) 99:1912–8. doi: 10.1097/TP.0000000000000598 25675205

[26] Love MI Huber W Anders S. Moderated estimation of fold change and dispersion for RNA-seq data with DESeq2. Genome Biol. (2014) 15:550. doi: 10.1186/s13059-014-0550-8 25516281 PMC4302049

[27] Ritchie ME Phipson B Wu D Hu Y Law CW Shi W. limma powers differential expression analyses for RNA-sequencing and microarray studies. Nucleic Acids Res. (2015) 43:e47. doi: 10.1093/nar/gkv007 25605792 PMC4402510

[28] Zou Y Xie J Zheng S Liu W Tang Y Tian W. Leveraging diverse cell-death patterns to predict the prognosis and drug sensitivity of triple-negative breast cancer patients after surgery. Int J Surg. (2022) 107:106936. doi: 10.1016/j.ijsu.2022.106936 36341760

[29] Newman AM Liu CL Green MR Gentles AJ Feng W Xu Y. Robust enumeration of cell subsets from tissue expression profiles. Nat Methods. (2015) 12:453–7. doi: 10.1038/nmeth.3337 PMC4739640 25822800

[30] Peng S Chen M Yin M Feng H. Identifying the potential therapeutic targets for atopic dermatitis through the immune infiltration analysis and construction of a ceRNA network. Clin Cosmet Investig Dermatol. (2021) 14:437–53. doi: 10.2147/CCID.S310426 PMC8112859 33994801

[31] Shen L Zhou K Liu H Yang J Huang S Yu F. Prediction of mechanosensitive genes in vascular endothelial cells under high wall shear stress. Front Genet. (2021) 12:796812. doi: 10.3389/fgene.2021.796812 35087573 PMC8787366

[32] Ide S Kobayashi Y Ide K Strausser SA Abe K Herbek S. Ferroptotic stress promotes the accumulation of pro-inflammatory proximal tubular cells in maladaptive renal repair. Elife. (2021) 10:e68603. doi: 10.7554/eLife.68603 34279220 PMC8318592

[33] Butler A Hoffman P Smibert P Papalexi E Satija R. Integrating single-cell transcriptomic data across different conditions, technologies, and species. Nat Biotechnol. (2018) 36:411–20. doi: 10.1038/nbt.4096 PMC6700744 29608179

[34] Korsunsky I Millard N Fan J Slowikowski K Zhang F Wei K. Fast, sensitive and accurate integration of single-cell data with Harmony. Nat Methods. (2019) 16:1289–96. doi: 10.1038/s41592-019-0619-0 PMC6884693 31740819

[35] Zheng L Qin S Si W Wang A Xing B Gao R. Pan-cancer single-cell landscape of tumor-infiltrating T cells. Science. (2021) 374:abe6474. doi: 10.1126/science.abe6474 34914499

[36] Szegezdi E Logue SE Gorman AM Samali A. Mediators of endoplasmic reticulum stress-induced apoptosis. EMBO Rep. (2006) 7:880–5. doi: 10.1038/sj.embor.7400779 PMC1559676 16953201

[37] Zhang K Kaufman RJ. From endoplasmic-reticulum stress to the inflammatory response. Nature. (2008) 454:455–62. doi: 10.1038/nature07203 PMC2727659 18650916

[38] De Chiara L Conte C Semeraro R Diaz-Bulnes P Angelotti ML Mazzinghi B. Tubular cell polyploidy protects from lethal acute kidney injury but promotes consequent chronic kidney disease. Nat Commun. (2022) 13:5805. doi: 10.1038/s41467-022-33110-5 36195583 PMC9532438

[39] Wang Y Li Y Chen Z Yuan Y Su Q Ye K. GSDMD-dependent neutrophil extracellular traps promote macrophage-to-myofibroblast transition and renal fibrosis in obstructive nephropathy. Cell Death Dis. (2022) 13:693. doi: 10.1038/s41419-022-05138-4 35941120 PMC9360039

[40] Rudman-Melnick V Adam M Potter A Chokshi SM Ma Q Drake KA. Single-cell profiling of AKI in a murine model reveals novel transcriptional signatures, profibrotic phenotype, and epithelial-to-stromal crosstalk. J Am Soc Nephrol. (2020) 31:2793–814. doi: 10.1681/ASN.2020010052 PMC7790221 33115917

[41] Nieuwenhuijs-Moeke GJ Pischke SE Berger SP Sanders JSF Pol RA Struys MMRF. Ischemia and reperfusion injury in kidney transplantation: relevant mechanisms in injury and repair. J Clin Med. (2020) 9:253. doi: 10.3390/jcm9010253 31963521 PMC7019324

[42] Inagi R. Endoplasmic reticulum stress as a progression factor for kidney injury. Curr Opin Pharmacol. (2010) 10:156–65. doi: 10.1016/j.coph.2009.11.006 20045381

[43] Fougeray S Bouvier N Beaune P Legendre C Anglicheau D Thervet E. Metabolic stress promotes renal tubular inflammation by triggering the unfolded protein response. Cell Death Dis. (2011) 2:e143. doi: 10.1038/cddis.2011.26 21490675 PMC3122058

[44] Tan X Yu L Yang R Tao Q Xiang L Xiao J. Fibroblast growth factor 10 attenuates renal damage by regulating endoplasmic reticulum stress after ischemia-reperfusion injury. Front Pharmacol. (2020) 11:39. doi: 10.3389/fphar.2020.00039 32116715 PMC7019113

[45] Badiola N Penas C Miñano-Molina A Barneda-Zahonero B Fadó R Sánchez-Opazo G. Induction of ER stress in response to oxygen-glucose deprivation of cortical cultures involves the activation of the PERK and IRE-1 pathways and of caspase-12. Cell Death Dis. (2011) 2:e149. doi: 10.1038/cddis.2011.31 21525936 PMC3122062

[46] Schonthaler HB Guinea-Viniegra J Wagner EF. Targeting inflammation by modulating the Jun/AP-1 pathway. Ann Rheum Dis. (2011) 70 Suppl 1:i109–112. doi: 10.1136/ard.2010.140533 21339212

[47] Hess J Angel P Schorpp-Kistner M. AP-1 subunits: quarrel and harmony among siblings. J Cell Sci. (2004) 117:5965–73. doi: 10.1242/jcs.01589 15564374

[48] Liu T Zhou Y Liu Y-C Wang J-Y Su Q Tang Z-L. Coronary microembolization induces cardiomyocyte apoptosis through the LOX-1-dependent endoplasmic reticulum stress pathway involving JNK/P38 MAPK. Can J Cardiol. (2015) 31:1272–81. doi: 10.1016/j.cjca.2015.01.013 26095939

[49] Nakagomi S Suzuki Y Namikawa K Kiryu-Seo S Kiyama H. Expression of the activating transcription factor 3 prevents c-Jun N-terminal kinase-induced neuronal death by promoting heat shock protein 27 expression and Akt activation. J Neurosci. (2003) 23:5187–96. doi: 10.1523/JNEUROSCI.23-12-05187.2003 PMC6741209 12832543

[50] Haber BA Mohn KL Diamond RH Taub R. Induction patterns of 70 genes during nine days after hepatectomy define the temporal course of liver regeneration. J Clin Invest. (1993) 91:1319–26. doi: 10.1172/JCI116332 PMC288102 8473485

[51] Liu Y Hu Y Xiong J Zeng X. Overexpression of activating transcription factor 3 alleviates cardiac microvascular ischemia/reperfusion injury in rats. Front Pharmacol. (2021) 12:598959. doi: 10.3389/fphar.2021.598959 33679395 PMC7934060

[52] Zhang K Shen X Wu J Sakaki K Saunders T Rutkowski DT. Endoplasmic reticulum stress activates cleavage of CREBH to induce a systemic inflammatory response. Cell. (2006) 124:587–99. doi: 10.1016/j.cell.2005.11.040 16469704

[53] Grootjans J Kaser A Kaufman RJ Blumberg RS. The unfolded protein response in immunity and inflammation. Nat Rev Immunol. (2016) 16:469–84. doi: 10.1038/nri.2016.62 PMC5310224 27346803

[54] McCord JM. Oxygen-derived free radicals in postischemic tissue injury. N Engl J Med. (1985) 312:159–63. doi: 10.1056/NEJM198501173120305 2981404

[55] Yellon DM Hausenloy DJ. Myocardial reperfusion injury. N Engl J Med. (2007) 357:1121–35. doi: 10.1056/NEJMra071667 17855673

[56] Qu K Shen N Xu X Su H Wei J Tai M. Emodin induces human T cell apoptosis *in vitro* by ROS-mediated endoplasmic reticulum stress and mitochondrial dysfunction. Acta Pharmacol Sin. (2013) 34:1217–28. doi: 10.1038/aps.2013.58 PMC4003158 23811723

[57] Liu Z-W Zhu H-T Chen K-L Dong X Wei J Qiu C. Protein kinase RNA-like endoplasmic reticulum kinase (PERK) signaling pathway plays a major role in reactive oxygen species (ROS)-mediated endoplasmic reticulum stress-induced apoptosis in diabetic cardiomyopathy. Cardiovasc Diabetol. (2013) 12:158. doi: 10.1186/1475-2840-12-158 24180212 PMC4176998

[58] Akhter N Wilson A Arefanian H Thomas R Kochumon S Al-Rashed F. Endoplasmic reticulum stress promotes the expression of TNF-α in THP-1 cells by mechanisms involving ROS/CHOP/HIF-1α and MAPK/NF-κB pathways. Int J Mol Sci. (2023) 24:15186. doi: 10.3390/ijms242015186 37894865 PMC10606873

[59] Pahl HL Baeuerle PA. The ER-overload response: activation of NF-kappa B. Trends Biochem Sci. (1997) 22:63–7. doi: 10.1016/s0968-0004(96)10073-6 9048485

[60] Hu P Han Z Couvillon AD Kaufman RJ Exton JH. Autocrine tumor necrosis factor alpha links endoplasmic reticulum stress to the membrane death receptor pathway through IRE1alpha-mediated NF-kappaB activation and down-regulation of TRAF2 expression. Mol Cell Biol. (2006) 26:3071–84. doi: 10.1128/MCB.26.8.3071-3084.2006 PMC1446932 16581782

[61] Hu H Tian M Ding C Yu S. The C/EBP homologous protein (CHOP) transcription factor functions in endoplasmic reticulum stress-induced apoptosis and microbial infection. Front Immunol. (2018) 9:3083. doi: 10.3389/fimmu.2018.03083 30662442 PMC6328441

[62] Yang J-R Yao F-H Zhang J-G Ji Z-Y Li K-L Zhan J. Ischemia-reperfusion induces renal tubule pyroptosis via the CHOP-caspase-11 pathway. Am J Physiol Renal Physiol. (2014) 306:F75–84. doi: 10.1152/ajprenal.00117.2013 24133119

[63] Liu H Chen Z Weng X Chen H Du Y Diao C. Enhancer of zeste homolog 2 modulates oxidative stress-mediated pyroptosis *in vitro* and in a mouse kidney ischemia-reperfusion injury model. FASEB J. (2020) 34:835–52. doi: 10.1096/fj.201901816R 31914694

[64] Tajima T Yoshifuji A Matsui A Itoh T Uchiyama K Kanda T. β-hydroxybutyrate attenuates renal ischemia-reperfusion injury through its anti-pyroptotic effects. Kidney Int. (2019) 95:1120–37. doi: 10.1016/j.kint.2018.11.034 30826015

[65] Diao C Chen Z Qiu T Liu H Yang Y Liu X. Inhibition of PRMT5 attenuates oxidative stress-induced pyroptosis via activation of the Nrf2/HO-1 signal pathway in a mouse model of renal ischemia-reperfusion injury. Oxid Med Cell Longev. (2019) 2019:2345658. doi: 10.1155/2019/2345658 31885778 PMC6899313

[66] Pang Y Zhang P-C Lu R-R Li H-L Li J-C Fu H-X. Andrade-Oliveira Salvianolic acid B modulates caspase-1-mediated pyroptosis in renal ischemia-reperfusion injury via Nrf2 pathway. Front Pharmacol. (2020) 11:541426. doi: 10.3389/fphar.2020.541426 33013384 PMC7495093

[67] Bando Y Tsukamoto Y Katayama T Ozawa K Kitao Y Hori O. ORP150/HSP12A protects renal tubular epithelium from ischemia-induced cell death. FASEB J. (2004) 18:1401–3. doi: 10.1096/fj.03-1161fje 15240565

[68] Fu Z-J Wang Z-Y Xu L Chen X-H Li X-X Liao W-T. HIF-1α-BNIP3-mediated mitophagy in tubular cells protects against renal ischemia/reperfusion injury. Redox Biol. (2020) 36:101671. doi: 10.1016/j.redox.2020.101671 32829253 PMC7452120

[69] Smith SF Hosgood SA Nicholson ML. Ischemia-reperfusion injury in renal transplantation: 3 key signaling pathways in tubular epithelial cells. Kidney Int. (2019) 95:50–6. doi: 10.1016/j.kint.2018.10.009 30606429

[70] Chen GY Nuñez G. Sterile inflammation: sensing and reacting to damage. Nat Rev Immunol. (2010) 10:826–37. doi: 10.1038/nri2873 PMC3114424 21088683

[71] Devarajan P. Update on mechanisms of ischemic acute kidney injury. J Am Soc Nephrol. (2006) 17:1503–20. doi: 10.1681/ASN.2006010017 16707563

[72] Awad AS Rouse M Huang L Vergis AL Reutershan J Cathro HP. Compartmentalization of neutrophils in the kidney and lung following acute ischemic kidney injury. Kidney Int. (2009) 75:689–98. doi: 10.1038/ki.2008.648 PMC2656389 19129795

[73] Kelly KJ Williams WW Colvin RB Meehan SM Springer TA Gutierrez-Ramos JC. Intercellular adhesion molecule-1-deficient mice are protected against ischemic renal injury. J Clin Invest. (1996) 97:1056–63. doi: 10.1172/JCI118498 PMC507153 8613529

[74] Kelly KJ Williams WW Colvin RB Bonventre JV. Antibody to intercellular adhesion molecule 1 protects the kidney against ischemic injury. Proc Natl Acad Sci USA. (1994) 91:812–6. doi: 10.1073/pnas.91.2.812 PMC43039 7904759

[75] Salmela K Wramner L Ekberg H Hauser I Bentdal O Lins LE. A randomized multicenter trial of the anti-ICAM-1 monoclonal antibody (enlimomab) for the prevention of acute rejection and delayed onset of graft function in cadaveric renal transplantation: a report of the European Anti-ICAM-1 Renal Transplant Study Group. Transplantation. (1999) 67:729–36. doi: 10.1097/00007890-199903150-00015 10096530

[76] He L Wei Q Liu J Yi M Liu Y Liu H. AKI on CKD: heightened injury, suppressed repair, and the underlying mechanisms. Kidney Int. (2017) 92:1071–83. doi: 10.1016/j.kint.2017.06.030 PMC5683166 28890325

[77] Venkatachalam MA Weinberg JM Kriz W Bidani AK. Failed tubule recovery, AKI-CKD transition, and kidney disease progression. J Am Soc Nephrol. (2015) 26:1765–76. doi: 10.1681/ASN.2015010006 PMC4520181 25810494

[78] Basile DP Bonventre JV Mehta R Nangaku M Unwin R Rosner MH. Progression after AKI: understanding maladaptive repair processes to predict and identify therapeutic treatments. J Am Soc Nephrol. (2016) 27:687–97. doi: 10.1681/ASN.2015030309 PMC4769207 26519085

[79] Yan M Shu S Guo C Tang C Dong Z. Endoplasmic reticulum stress in ischemic and nephrotoxic acute kidney injury. Ann Med. (2018) 50:381–90. doi: 10.1080/07853890.2018.1489142 PMC6333465 29895209

[80] Chiang C-K Hsu S-P Wu C-T Huang J-W Cheng H-T Chang Y-W. Endoplasmic reticulum stress implicated in the development of renal fibrosis. Mol Med. (2011) 17:1295–305. doi: 10.2119/molmed.2011.00131 PMC3324175 21863214

[81] Maestri NE Brusilow SW Clissold DB Bassett SS. Long-term treatment of girls with ornithine transcarbamylase deficiency. N Engl J Med. (1996) 335:855–9. doi: 10.1056/NEJM199609193351204 8778603

[82] Collins AF Pearson HA Giardina P McDonagh KT Brusilow SW Dover GJ. Oral sodium phenylbutyrate therapy in homozygous beta thalassemia: a clinical trial. Blood. (1995) 85:43–9. doi: 10.1182/blood.V85.1.43.bloodjournal85143 7528572

[83] Chen WY Bailey EC McCune SL Dong JY Townes TM. Reactivation of silenced, virally transduced genes by inhibitors of histone deacetylase. Proc Natl Acad Sci USA. (1997) 94:5798–803. doi: 10.1073/pnas.94.11.5798 PMC20860 9159154

[84] Xu D Li W Zhang T Wang G. miR-10a overexpression aggravates renal ischemia-reperfusion injury associated with decreased PIK3CA expression. BMC Nephrol. (2020) 21:248. doi: 10.1186/s12882-020-01898-3 32611384 PMC7329557

[85] Hao J Wei Q Mei S Li L Su Y Mei C. Induction of microRNA-17-5p by p53 protects against renal ischemia-reperfusion injury by targeting death receptor 6. Kidney Int. (2017) 91:106–18. doi: 10.1016/j.kint.2016.07.017 PMC5179285 27622990

[86] Hu H Jiang W Xi X Zou C Ye Z. MicroRNA-21 attenuates renal ischemia reperfusion injury via targeting caspase signaling in mice. Am J Nephrol. (2014) 40:215–23. doi: 10.1159/000368202 25322693

[87] Liu X-J Hong Q Wang Z Yu Y-Y Zou X Xu L-H. MicroRNA-34a suppresses autophagy in tubular epithelial cells in acute kidney injury. Am J Nephrol. (2015) 42:168–75. doi: 10.1159/000439185 26406385

[88] Ke J Zhao F Luo Y Deng F Wu X. MiR-124 negatively regulated PARP1 to alleviate renal ischemia-reperfusion injury by inhibiting TNFα/RIP1/RIP3 pathway. Int J Biol Sci. (2021) 17:2099–111. doi: 10.7150/ijbs.58163 PMC8193263 34131409

[89] Chen H-H Lan Y-F Li H-F Cheng C-F Lai P-F Li W-H. Urinary miR-16 transactivated by C/EBPβ reduces kidney function after ischemia/reperfusion-induced injury. Sci Rep. (2016) 6:27945. doi: 10.1038/srep27945 27297958 PMC4906401

[90] Lorenzen JM Kaucsar T Schauerte C Schmitt R Rong S Hübner A. MicroRNA-24 antagonism prevents renal ischemia reperfusion injury. J Am Soc Nephrol. (2014) 25:2717–29. doi: 10.1681/ASN.2013121329 PMC4243358 24854275

[91] Wu X-Q Tian X-Y Wang Z-W Wu X Wang J-P Yan T-Z. miR-191 secreted by platelet-derived microvesicles induced apoptosis of renal tubular epithelial cells and participated in renal ischemia-reperfusion injury via inhibiting CBS. Cell Cycle. (2019) 18:119–29. doi: 10.1080/15384101.2018.1542900 PMC6343735 30394829

[92] Gong S Zhang A Yao M Xin W Guan X Qin S. REST contributes to AKI-to-CKD transition through inducing ferroptosis in renal tubular epithelial cells. JCI Insight. (2023) 8:e166001. doi: 10.1172/jci.insight.166001 37288660 PMC10393228

[93] Doncheva NT Palasca O Yarani R Litman T Anthon C Groenen MAM. Human pathways in animal models: possibilities and limitations. Nucleic Acids Res. (2021) 49:1859–71. doi: 10.1093/nar/gkab012 PMC7913694 33524155

[94] Bagul A Frost JH Drage M. Stem cells and their role in renal ischaemia reperfusion injury. Am J Nephrol. (2013) 37:16–29. doi: 10.1159/000345731 23295823

[95] Simona M-S Alessandra V Emanuela C Elena T Michela M Fulvia G. Evaluation of oxidative stress and metabolic profile in a preclinical kidney transplantation model according to different preservation modalities. Int J Mol Sci. (2023) 24:1029. doi: 10.3390/ijms24021029 36674540 PMC9861050

[96] Wei Q Dong Z. Mouse model of ischemic acute kidney injury: technical notes and tricks. Am J Physiol Renal Physiol. (2012) 303:F1487–1494. doi: 10.1152/ajprenal.00352.2012 PMC3532486 22993069

