# Supplementary material for: Comprehensive molecular and cellular characterization of endoplasmic reticulum stress-related key genes in renal ischemia/reperfusion injury
Source: Front Immunol. 2024 Mar 1;15:1340997. doi: 10.3389/fimmu.2024.1340997 (PMC10940334; doi:10.3389/fimmu.2024.1340997)
Supplement: Supplementary file 1 [file DataSheet_1.pdf]

Supplementary Material

1 Supplementary Figures and Tables

1.1 Supplementary Figures

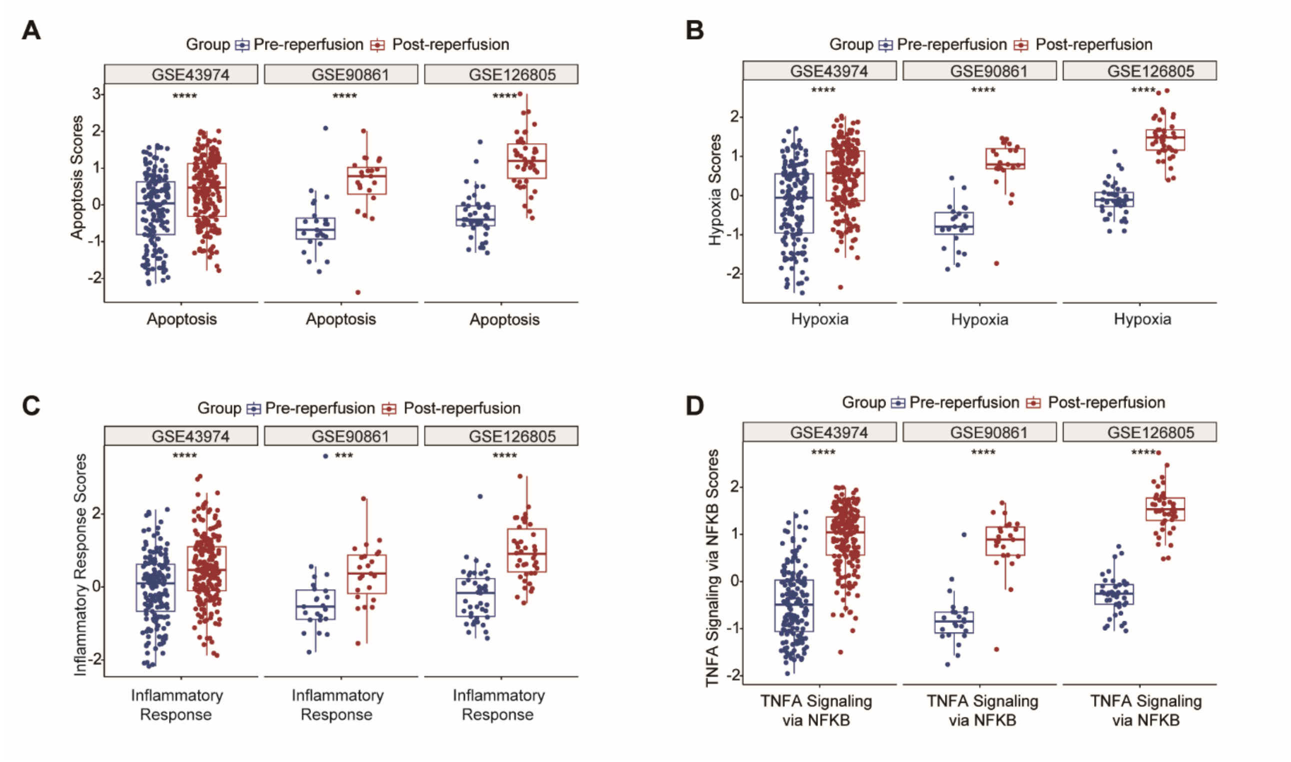

Supplementary Figure 1. Upregulated biological pathways during RIRI.

Boxplots comprising apoptosis (A), hypoxia (B), inflammatory response (C) and TNFA signaling via NF-kappa B (D) scores of pre- and post-reperfusion groups in GSE43974, GSE90861 and GSE126805. ***p<0.001, ****p<0.0001.

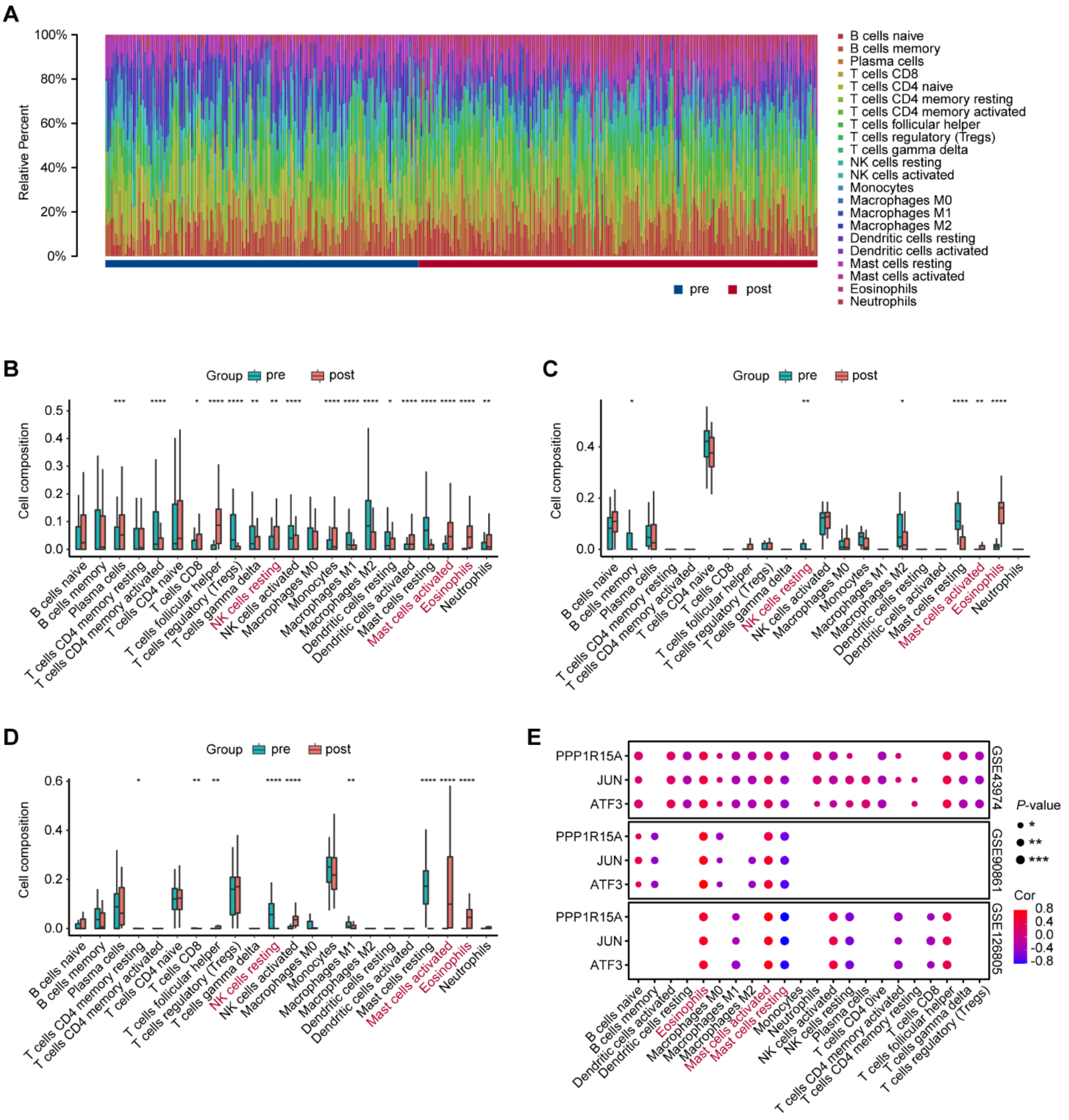

Supplementary Figure 2. Immune cell infiltration analyses for renal tissues of pre- and post-reperfusion.

A Relative proportion of immune infiltration abundances for each sample. B-D Box plots depicting that NK resting cells, mast activated cells and eosinophils are consistently significantly higher infiltrated in post-reperfusion samples in GSE43974 (B), GSE90861 (C) and GSE12680 (D). E Bubble plot showing relevance scores of 22 immune cells infiltrations and key ERS-related gene expression levels.

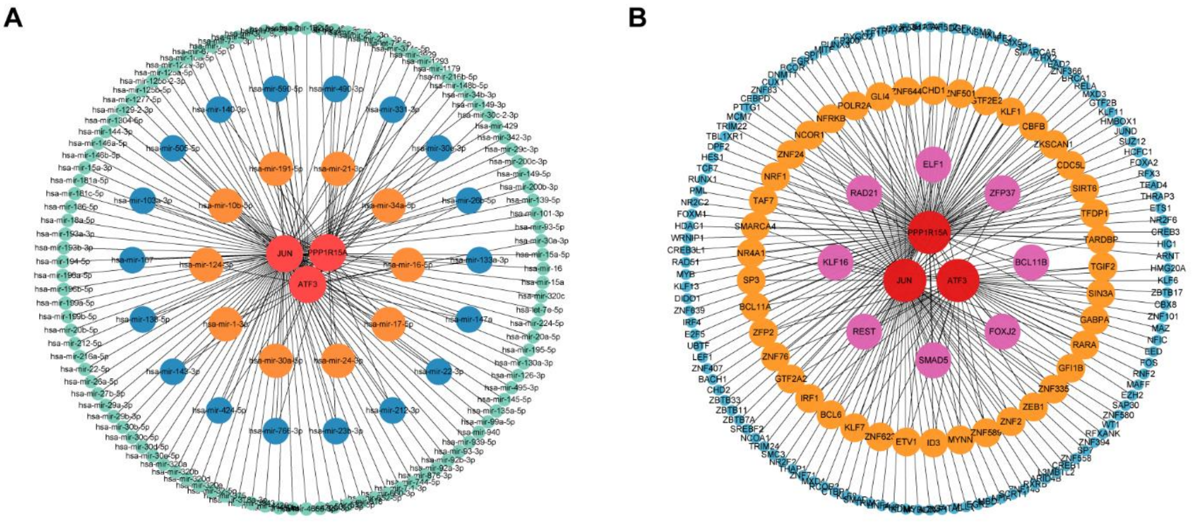

Supplementary Figure 3. The miRNA and TF-mRNA regulatory network for three hub genes.

The miRNA network (A) and TF network (B) of PPP1R15A, JUN and ATF3.

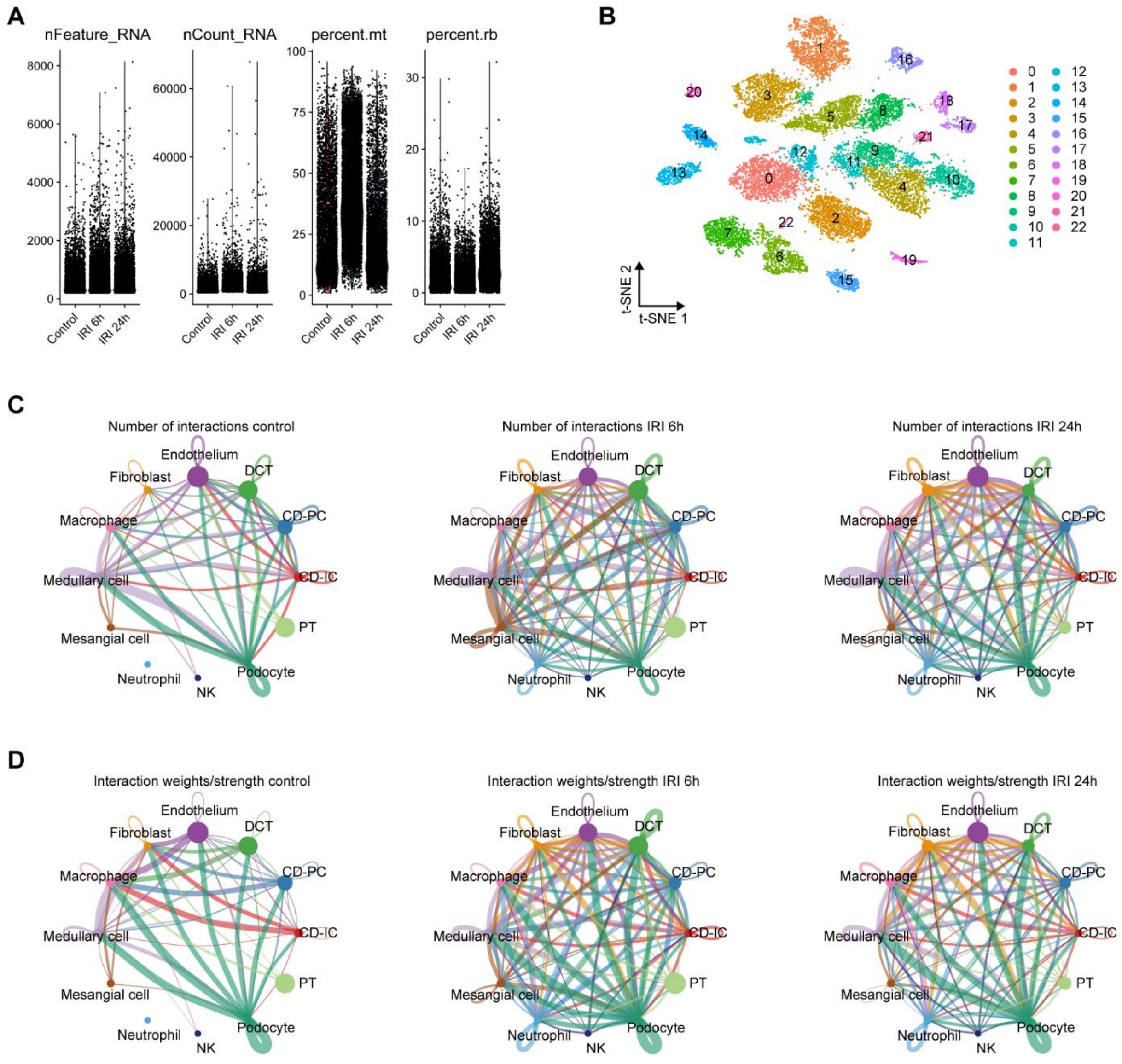

Supplementary Figure 4. Quality control and cell-chat analysis in kidneys during IRI.

A Quality control of single-cell RNA-seq data of control, IRI 6h and IRI 24h samples. B tSNE plot showing 16,658 cells from the sham control, IRI-6h and IRI-24h samples, colored by 22 clusters. C, D The inferred cellular networks. Circle sizes and edge width represents the number of cells in each cell type and number of cell-cell interactions or cell-cell interaction weights/strength for control, IRI 6h and IRI 24h samples.
